# Investigation of GPR137C as a promising novel marker for the progression of prostate cancer through G4 screen and bioinformatics analyses

**DOI:** 10.3389/fimmu.2025.1576835

**Published:** 2025-05-30

**Authors:** Yue Hou, Haowen Lu, Saisai Chen, Likai Mao, Xuan Huang, Feng Xu, Chuanjun Shu

**Affiliations:** ^1^ Military Medical Innovation Center, Fourth Military Medical University, Xi’an, China; ^2^ Department of Urology, Affiliated Zhongda Hospital of Southeast University, Nanjing, Jiangsu, China; ^3^ Reproductive Medical Center, Jinling Hospital Affiliated to Medical School of Nanjing University, Nanjing, Jiangsu, China; ^4^ Department of Urology, Jinhu County Peoples Hospital, Huai’an, Jiangsu, China; ^5^ Department of Bioinformatics, School of Biomedical Engineering and Informatics, Nanjing Medical University, Nanjing, China

**Keywords:** G-quadruplex, prostate cancer, GPR137C, GPCRs de-orphanization, drugs

## Abstract

**Introduction:**

Prostate cancer (PCa) remains the fifth leading cause of male cancer mortality, necessitating novel biomarkers and therapeutic targets.

**Methods:**

Through BG4 ChIP-seq profiling in PCa cells, we identified promoter G-quadruplex (G4) structures in prognosis-associated genes, with GPR137C exhibiting a functional G4 in its promoter.

**Results:**

This G4 structure facilitates promoter hypomethylation to activate GPR137C transcription. Moreover, GPR137C promotes tumor microenvironment remodeling by enhancing immune cell infiltration, thereby driving PCa progression.

**Disscussion:**

This study establishes promoter G4s as epigenetic regulators in PCa while proposing GPR137C as both a prognostic biomarker and a therapeutic nexus for GPCR-targeted drug development.

## Introduction

Prostate cancer is a complex disease that affects millions of men globally, predominantly in high human development index regions (1, 2). However, while some types of prostate cancer grow slowly and may need minimal or even no treatment, other types are aggressive and can spread quickly (3, 4). Tumors that have metastasized to distant body sites are most dangerous, with 5-year survival rates of 30%–40% (5, 6). However, no drug or vaccine is approved by regulatory agencies for the prevention of prostate cancer (7, 8). Each available treatment options for Prostate adenocarcinoma (PRAD) patients were closely associated with severe side effects, i.e., toxicity and reduced white and red blood cell counts, hair loss, fatigue, erectile incontinence, and so on (1, 9). Therefore, the discovery of novel drug targets to effectively resist prostate cancer is urgently needed.

G-protein coupled receptors (GPCRs) are the largest and most versatile cell surface receptor family with a broad repertoire of ligands and functions (10, 11). They are the largest targets in approved drugs (12). However, currently established GPCR drug targets are widely utilized by distinct approved agents (13, 14). A very few GPCR-related drugs have been effectively exploited in pursuit of anti-cancer therapies (15). Hence, orphan GPCR (oGPCR), which endogenous ligands have not yet been identified, is a good option for candidate indictors/drugs in detection/therapy for PRAD patients (16).

The utilization of oGPCRs in drugs is limited due to their unknown mechanism of activation or de-orphanization (15, 17–19). However, four-stranded DNA G-quadruplex (G4) structures are important features of transcriptional regulation that coordinate recruitment of key chromatin proteins and the transcriptional machinery through interactions with DNA secondary structure, rather than primary sequence (20, 21). Previous studies indicated that interactions exist between transcription factors and G4 structures at gene promoters, especially oncogene such as MYC and SP1 (22, 23). These results suggested that G4 structures, which are implicated in transcriptional regulation of oncogene in many cancers, could act as a promising target for cancer therapy (21). Hence, G4 and GPCRs could be combined to be utilized in therapy of cancer patients. However, the relationship between G4 and prognosis-related oGPCRs in PRAD are unknown. Therefore, G4 structures were screened in PRAD prognosis-related oGPCRs in this study.

Here, G4 structures in prostate cancer cells (C4-2) were systemically annotated, and the prognostic values of their related genes in patients were analyzed. Then, GPR137C was found not only to be a prognosis-related oGPCR gene in prostate cancer but also has a G4 structure in its promoter. While preliminary investigations into GPR137C’s oncological roles have concentrated on small-cell lung cancer, initial observations indicate that its expression dynamics could serve as a potential prognostic biomarker in this malignancy (24). Meanwhile, GPR137C functions and the cause of expression change in PRAD are unknown. In this study, G4 structure probably resulted in the hypomethylation of the promoter and then led to transcriptional activation of GPR137C. Moreover, GPR137C was highly expressed in patients, and greater expression was linked to worse prognosis. G4 drives oncogenic GPR137C to promote infiltration levels in cancer-related cells and then to contribute to the malignant progression of prostate cancer. Furthermore, acetaminophen, valproic acid, and MPIX-(PEG) 2-G (PNA)-d-Lys-d-Lys (mPG) probably could be utilized to target GPR137C to inhibit the development of PRAD in patients. Hence, GPR137C probably is a good druggable receptor and biomarker for prostate cancer. Our results may provide new insight into the druggable receptor identification and oGPCRs de-orphanization, and G4 in the promoter may benefit prostate cancer patients as a novel early genetic indicator.

## Materials and methods

### Cell lines and culture

Human prostate cancer cell line (C4-2) was cultured in RPMI 1640 medium (Gibco Thermo Fisher Scientific, USA), containing 10% fetal bovine serum (LONSERA, Uruguay) and 1% penicillin–streptomycin solution (Keygen, China). All cell lines were purchased from the American Type Culture Collection (ATCC, USA) and incubated in 95% humidified air at 37°C and 5% CO_2_.

### BG4 ChIP-seq

BG4 ChIP experiment was preformed based on a published protocol with minor adaptations (25). C4–2 cells were crosslinked with 1% formaldehyde for 10 min at RT and stopped by adding 125 mM glycine for 10 min. After washing twice with ice-cold PBS, the cells were pellet down at 300 g for 5 min at 4°C. The cell pellets were resuspended in 1 ml of ice-cold cyto-lysis buffer and incubated on ice for 10 min with occasional inversion every 2 min. The cells were pellet down at 2,300 *g* for 5 min at 4°C. The nuclear lysate was then sonicated seven times. The soluble chromatin was collected by centrifugation at 14,000 rpm for 10 min. One µg of BG4 antibody was added to the mixture and incubated by rotation at 1,400 rpm in a thermomixer at 16°C for 1 h. To pull down BG4-bound chromatin fragments, 10 μl of pre-blocked anti-FLAG M2 magnetic beads was applied to the tube and incubated at 16°C for 1 h. The beads were washed three times and then washed with the wash buffer for additional three times at 1,400 rpm for 10 min at 37°C. G4 DNA were eluted from the beads by adding 100 μl of TE buffer and 2 μl of proteinase K to the tube and incubated at 37°C for 2 h at 1,400 rpm, followed by de-crosslinking overnight at 65°C. To minimize potential PCR bias during library preparation, we employed 10 ng of input DNA for tagmentation reactions followed by standard Nextera barcoding and amplification with Q5 high-fidelity DNA polymerase (NEB) for both ChIP and input libraries. All libraries were sequenced to achieve >0.7× sequence coverage of the human genome per replicate, with four biological replicates of BG4 ChIP-seq performed in C4–2 cells to ensure experimental reproducibility.

### Sequencing alignment and processing

Human genome sequence build 38 (hg38) downloaded from the UCSC genome browser was used as the reference for mapping. All qualified sequencing reads were mapped to hg38 using BWA-MEM (version 0.7.12-r1039) in paired-end mode. The resulting SAM files were converted into BAM format, sorted based on genome position, and indexed using SAMtools (version 1.8).

### Transwell assay

In Transwell assay, cells were inoculated into a 24-well Transwell cell apical chamber containing matrix gel (BD, USA) to evaluate migration. The bottom and upper chambers contained the RPMI medium and serum-free medium, respectively. Cells that invaded the bottom chambers were fixed with 4% polyformaldehyde, stained with 0.1% crystal violet solution, and then photographed under a microscope.

### Small interfering RNA

The small interfering RNAs (siRNAs) of GPR137C was designed and synthesized by GenePharma Co. (China). The sequences of siRNA were set as follows: GGCGGAGGUUAUAUGUAAATT, GGCUCAUGUAGAAGACAUATT (siRNA for GPR137C sense), UUUACAUAUAACCUCCGCCTT, UAUGUCUUCUACAUG AGCCTT (siRNA for GPR137C anti-sense), UUCUCCGAACGUGUCACGUTT (ncRNA for GRDPH sense), and ACGUGACACGUUCGGAGAATT (ncRNA for GRDPH anti-sense).

### Dual-Luciferase Reporter Assay

The construction of referent and variant reporter plasmids was based on the pGL3-promoter vector. A 435-bp promoter fragment containing predictive G4-forming sequences was amplified from C4–2 cell genomic DNA by polymerase chain reaction (PCR). The variant reporter plasmid was created by overlap PCR. All the constructed plasmids were validated by sequencing. C4–2 cells were harvested at 48 h after transfection and investigated for luciferase activity using the Dual-Luciferase Reporter Assay System (Promega, Fitchburg, MA). Luciferase activity was normalized by phRL-TK luciferase signals.

### TCGA data download, process, and analysis

The mRNA expression data and clinical data of prostate cancer patients were downloaded from TCGA database (https://genome-cancer.ucsc.edu/). These expression data were first normalized, and differential expression analysis was then performed for SEMA3F by R package limma (26). The p-value <0.05 was considered as statistically significant. According to clinical data, we estimated cumulative survival curves and overall survival rates using KaplanߛMeier curves. Then, hazard ratio (HR) and corresponding 95% CIs were estimated based on Cox proportional hazard models. Indeed, higher HR values (HR > 1.0) indicate bad prognosis, while lower HR values (HR < 1.0) indicate good prognosis. The p-value was calculated by log-rank test. The expression associations between the genes of our interest were performed by Spearman’s correlation analysis. p < 0.05 represents statistical significance.

The mutation and copy number variations of TCGA-PRAD were compiled by using cBioPortal (27). Transcript expression of GPR137C in prostate and corresponding cancers were explored based on data of GTEx and TCGA database. The GPR137C protein level and tissue staining were explored based on Human Protein Atlas (HPA) database. Furthermore, mRNA alternative splicing situation for GPR137C was explored based on TCGA splicing variants database (TSVdb).

Furthermore, we downloaded a standardized pan-cancer dataset from the UCSC (https://xenabrowser.net/) database. Then, tumor mutational burden (TMB) was calculated by TMB function in R package mafTools for pan-cancer. Combined with expression of GPR137C in each cancer, the person correlation coefficient between GPR137C expression [log2(x+0.001) transformation] and neoantigen (NEO)/loss of heterozygosity (LOH)/homologous recombination deficiency (HRD)/ploidy/differentially methylated probes-based (DMPss), DNA methylation-based (DNAss)/enhancer elements/DNA methylation-based (ENHss) were computed, respectively.

### DNA methylation analysis

TCGA DNA methylation data files for GPR137C in prostate cancer and matched normal tissues were collected from Genomic Data Commons. We utilized the Illumina Human Methylation 450k R annotation data package to map the Illumina methylation array probes to individual genes. We then retained those probed mapped to the corresponding promoter region. Median beta values were utilized when genes had multiple probes. We then calculated median beta value for GPR137C in each sample to calculate the overall methylation level in the promoter region. To examine the regulation of GPR137C expression by DNA methylation, we estimated the Spearman correlation between DNA methylation beta values with mRNA expression for GPR137C.

### Functional enrichment analysis

The expression of related genes for GPR137C was calculated based on expression and Pearson’s correlation coefficient. The expression was downloaded from TCGA-PRAD and GTEx-prostate tissues. The GO and KEGG analyses for related genes were performed based on KOBAS (28).

### Immune infiltrate analysis

Infiltration levels for distinct immune cells in PRAD were quantified by using CIBERSORT and TIMER (29–31). CIBERSORT is an analytical tool, which was developed to provide an estimation of the abundances of member cell types in a mixed cell population based on gene expression data. Meanwhile, the TIMER is another tool for comprehensive analysis of tumor-infiltrating immune cells. Here, they were combined to analyze the correlation of GPR137C expression level with immune cell infiltration level in PRAD. p-value <0.05 was considered as statistically significant.

### Analysis of scRNA-seq data

The single-cell RNA sequencing data were downloaded from NCBI GEO database (GSE137829, GSE141445, GSE172301, and GSE176031) (32–35). The scRNA-seq data analysis was consistent with a previous study. The Seurat function “FindVariableFeatures” was first utilized to identify the highly variable genes (HVGs). Then, the top 2000 HVGs were applied for data integration. The data were scaled using “ScaleData,” and the first 40 principle components were chosen for auto-clustering analyses using “FindNeighbors” and “FindClusters” functions. For all cells, we identified clusters setting the resolution as 1.5. The clustering results were then visualized with the UMAP scatter plot. The marker genes of macrophage were downloaded from CellMark database (36).

### Structural pharmacological analysis

GPR137C 3D structure was predicted by AlphaFold3 (37). The ligand binding pockets of GPR137C were obtained by POCASA software (38). The interacting chemicals for GPCRs were calculated based on comparative toxicogenomics database (39). The complex of GPCRs and molecules were predicted by SwissDock software (40). The interactions between protein and molecule were identified based on the distance between two atoms.

### Statistical analysis

Data were analyzed using GraphPad Prism 8. Statistical significance (p < 0.05) between the means of two groups was determined using the Dunnett’s t-test. The difference in survival between two groups were calculated using the log-rank test. p < 0.05 was considered statistically significant.

## Results

### Screen the G4 profile of promoters in PRAD prognosis-associated GPCRs

**Figure 1:**
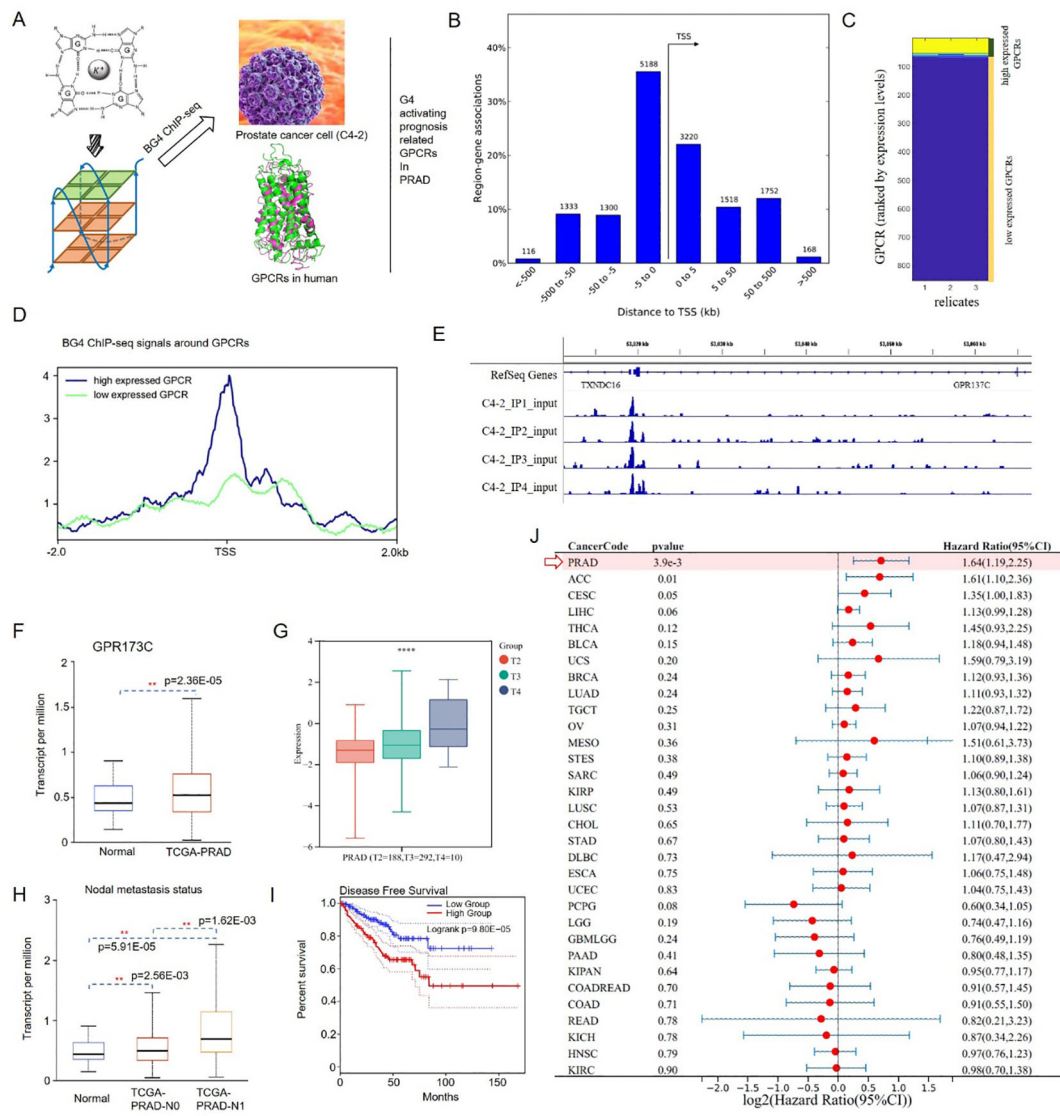
G4 activation PRAD prognosis-related gene GPR137C. **(A)** G4 screening process for oGPCRs in PRAD. **(B)** Gene association regions for G4 structures. **(C)** Low/high expression level in PRAD. **(D)** BG4 ChIP-seq signals around GPCRs in the two expression groups. **(E)** G4 structure in GPR137C promoter. **(F)** Transcript levels of GPR137C in normal and PRAD tissues. GPR137C expression level in different T stages **(G)** and nodal metastasis statuses **(H)**. **(I)** Disease-free survival plot for GPR137C in PRAD. **(J)** HR values of GPR137C for pan-cancer ** and **** represent p <0.05 and p< 0.0001.

G4 formation can affect gene regulation and has been associated with cancer progression. Then, G4 structures in prostate cancer cells (C4-2) were sequenced (**Figure 1A**). We found ∼10,000 G4 structures in chromatin, predominantly in the promoters and 5' UTRs of highly transcribed genes (**Figure 1B**). Meanwhile, GPCRs control many aspects of cancer progression including tumor growth, invasion, migration, survival, and metastasis. Furthermore, GPCRs activity can be altered in cancer through aberrant overexpression, especially orphan GPCRs (oGPCRs). Here, G4 formation in promoter could be utilized to explain aberrant overexpression of oGPCRs in PRAD cells (**Figures 1C, D**). By utilizing G4 and prognosis screen, we obtained an oGPCR, i.e., GPR137C (**Figure 1E**). A G4 ChIP-seq peak was presented in the promoter of GPR137C (**Figure 1E**). In addition, the construction of referent and variant reporter plasmids was based on the pGL3-promoter vector. A 435-bp promoter fragment containing G4-forming sequences (mutant and wide type) was amplified from C4–2 cell genomic DNA by polymerase chain reaction. Then, the luciferase results indicated that G4 in promoter enhances the expression level of GPR137C (**Supplementary Figure S1**). These results suggested that G4 in promoter probably act as a novel activating pathway for oGPCRs.

GPR137C was identified, as it not only is prognosis-related oGPCR in PCa, but it also has a G4 ChIP-seq peak in the promoter. According to TCGA database, GPR137C was a significance difference oGPCRs in PRAD and corresponding normal tissues (**Figure 1F**). Based on the clinical information for the TCGA-PRAD cohort, GPR137C expression level was then found to be positively correlated with pathological stage (T stage) and nodal metastasis status of PCa patients, respectively (**Figures 1G, H**). Meanwhile, low GPR137C expression was significantly correlated with better disease-free survival in PCa patients when compared to high expression group (p = 9.80E−05) (**Figure 1I**). Furthermore, GPR137C had the highest HR value in PCa patients when it was compared to pan-cancer based on forest plot (HR = 1.64, p = 3.9E−03) (**Figure 1J**). In addition, it was found that GPR137C could promote migration of prostate cancer cells (C4–2 and PC3) better when compared to the Transwell assays results for si-GPR137C and wild type (**Supplementary Figure S2**). These results indicated that GPR137C could probably act as novel biomarker for PRAD.

### G4 negatively correlated with methylation levels in promoter region of GPR137C in PRAD

**Figure 2:**
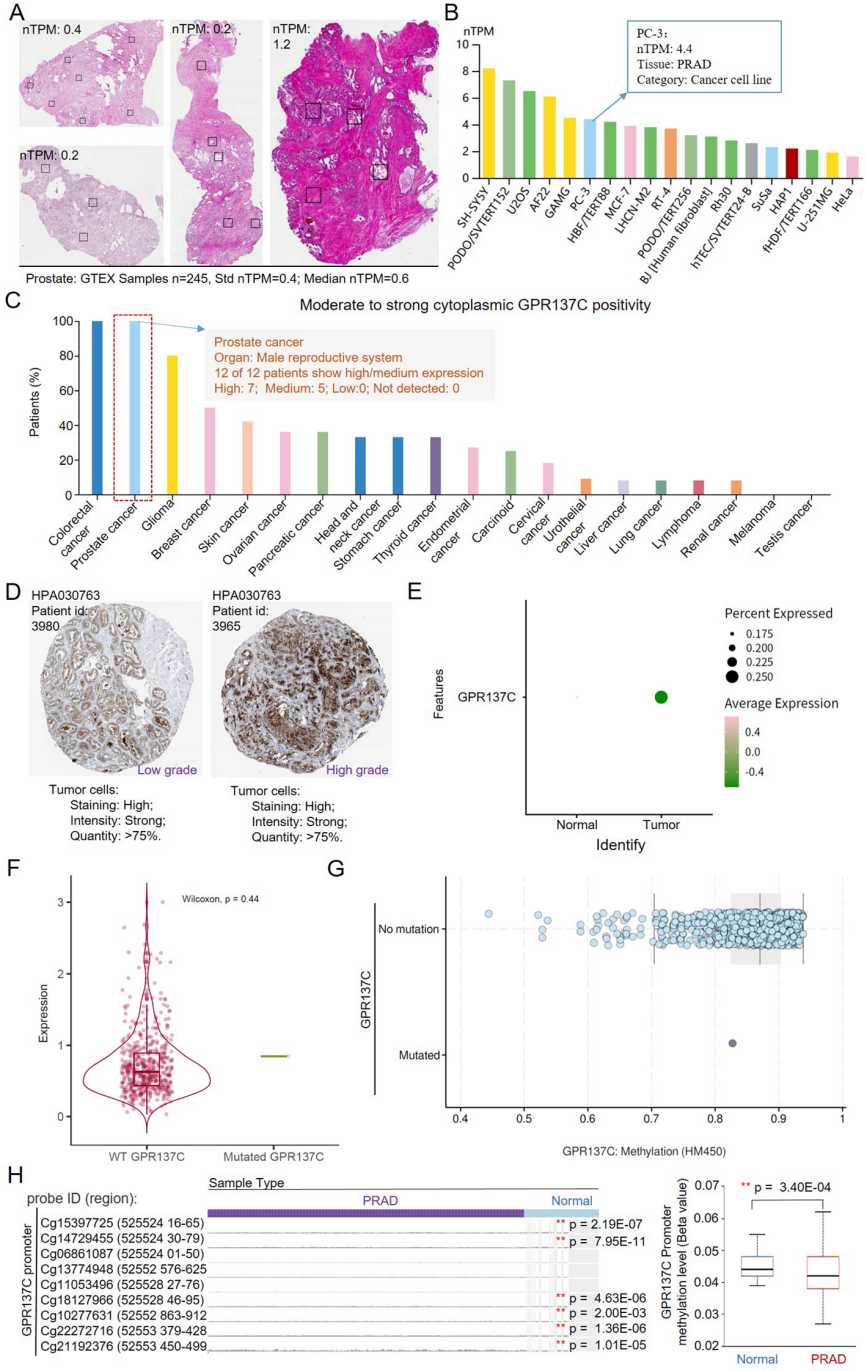
The G4 drives promoter hypomethylation in GPR137C. **(A)** GPR137C expression in normal prostate tissues. **(B)** GPR137C expression level in common cell lines. **(C)** The percent of moderate to strong cytoplasmic GPR137C positivity in stained cancer tissues. **(D)** The GPR137C expression level in different grades of PRAD tissues. **(E)** GPR137C expression level in normal and PRAD based on single-cell sequencing data. **(F)** GPR137C expression level in wild type and mutated group. **(G)** The methylation level in wild type and mutated group for GPR137C. **(H)** The methylation level in the promoter of GPR137C. ** represents p < 0.05.

According to HPA and GETX database, the average RNA expression for GPR137C in prostate was 0.5 and 0.6 nTPM, respectively (**Figure 2A**). However, the expression in prostate cancer cell (PC-3) for GPR137C was 4.4 nTPM, which was far high than that in normal prostate (**Figure 2B**; **Supplementary Figure S3**). Meanwhile, GPR137C protein was identified in 100% prostate cancer tissues (antibody, HP1030763) (**Figure 2C**). Moderate to strong cytoplasmic positivity for GPR137C was observed in prostate cancer (high, 58.33%; medium, 41.67%). There was no weak or negative stain for GPR137C in prostate cancer. Moreover, strong cytoplasmic positivity for GPR137C was both identified in low and high grade for PRAD patients, but most of it was distributed in high grade (**Figure 2D**; **Supplementary Figure S4**). These results indicated that protein expression for GPR137C was a potential biomarker and progress-related oGPCR for prostate cancer.

Single-cell sequencing data set GSE141445 was further utilized to compare GPR137C expression level in PRAD and corresponding normal tissues. Then, it was found that GPR137C was not only highly expressed in tumor samples but also has high percent expression when it was compared to normal samples (**Figure 2E**). Then, point mutations and methylation level for GPR137C were utilized to explain aberrant overexpression of GPR137C in PRAD tissues. It was found that point mutations in GPR137C could not affect the expression and methylation level of GPR137C in PRAD tissues (**Figures 2F, G**). However, methylation level in the promoter could affect the expression of GPR137C in PRAD patients. Low methylation level in the promoter was significantly correlated to high GPR137C expression in PRAD patients (p = 3.40E−04) (**Figure 2H**). Hence, G4 has a negative relationship with methylation level in the promoter for GPR137C. In details, based on a previous study (41), the G4 structure probably isolate the DNA methyltransferase Dnmt3a catalytic domain (DNMT3A-CD) and prevents its proper binding to DNA, resulting in hypomethylation, which leads to transcriptional activation of GPR137C.

### ENST00000321662.10 is a main cancer-progressed isoform for GPR137C in PRAD

**Figure 3:**
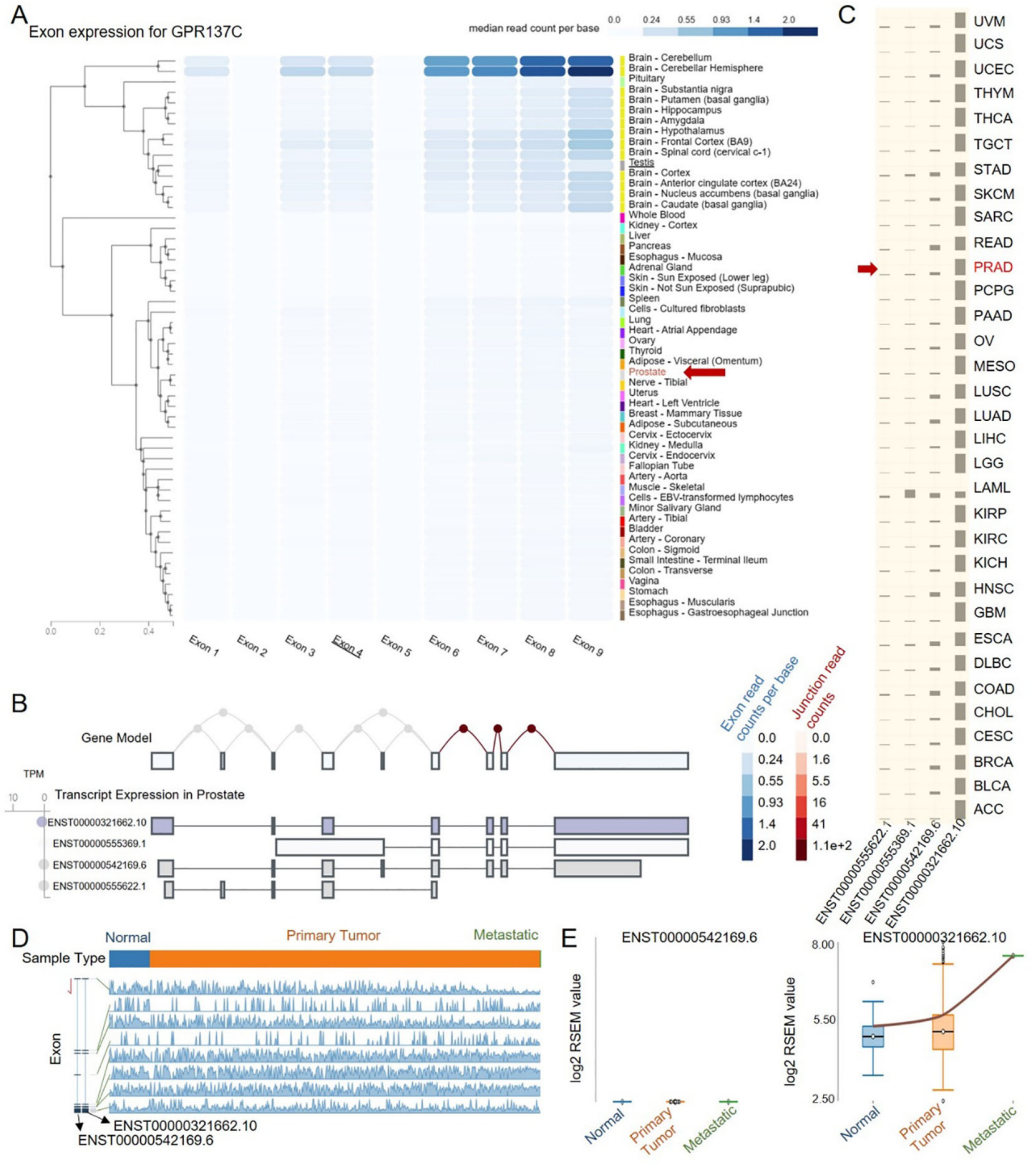
ENST00000321662.10 is a cancer-progress-related isoform for GPR137C in PRAD. **(A)** Exon expression for GPR137C in normal tissues. Transcript expression in prostate **(B)** and PRAD tissues **(C)**. **(D)** GPR137C-related isoform expression in primary tumor, metastatic, and corresponding normal tissues. **(E)** The RNA-Seq by expectation maximization (RSEM) value for two main isoforms of GPR137C in different tissues for TCGA-PRAD.

Isoforms are often tissue-specific and may alter the function, cellular localization, and stability of the corresponding RNA or protein. Meanwhile, alternative usage of transcript isoforms from the same gene has been hypothesized as an important feature in prostate cancer. Then, isoforms for GPR137C in normal and PRAD were explored. Based on the landscape of exon expression for GPR137C in GTEx database, it was found that GPR137C was enriched in the brain and testis but not in the prostate (**Figure 3A**). The main transcripts for GPR137C in prostate was ENST00000542169.6, ENST00000555622.1, and ENST00000321662.10 (**Figure 3B**). However, it was highly expressed in prostate cancer tissues. The main transcripts for GPR137C in TCGA-PRAD were ENST00000542169.6 and ENST00000321662.10 (**Figures 3C, D**).

From the landscape of transcript expression in prostate cancer and corresponding adjacent normal tissues, ENST00000321662.10 expression far exceeds other isoforms (**Figures 3B, D**). Meanwhile, the isoform ENST00000542169.6 was not only with a low expression in PCa tissues but also the expression presents no difference among normal, primary tumor, and metastatic cancer tissues. However, the isoform ENST00000321662.10 was not only with a high expression in PCa tissues but also the expression presents a significant difference among normal, primary tumor, and metastatic cancer tissues. The expression of the isoform ENST00000321662.10 gradually increases as the cancer progresses (**Figures 3D, E**). These results indicated that ENST00000321662.10 was a main cancer-progress-related isoform for GPR137C in PRAD.

### Functional roles of GPR137C in prostate cancer progress

**Figure 4:**
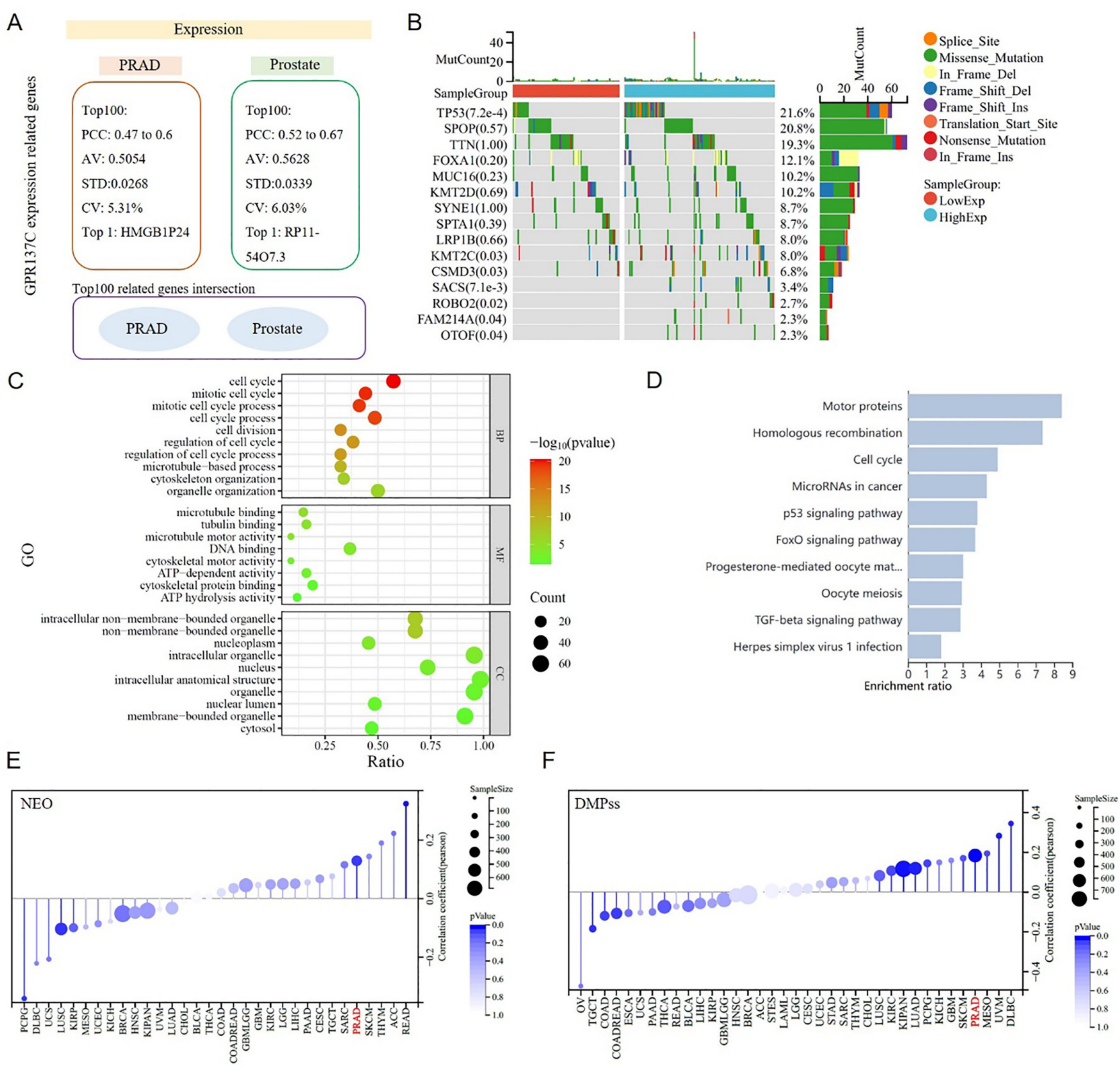
GPR137C function roles in PRAD. **(A)** GPR137C-related genes in prostate and TCGA-PRAD. No intersection sets between two top 100 related gene lists. **(B)** Top 15 GPR137C expression-related mutation genes in TCGA-PRAD. The enriched Gene Ontology (GO) items **(C)** and KEGG pathways **(D)** for top 100 GPR137C-related genes in TCGA-PRAD. The relationship between GPR137C expression and NEO (neoantigen) **(E)**/differentially methylated probes-based (DMPss) **(F)**. Both correlation coefficient values were more that 0.1 with a corresponding p-value < 0.05.

To explore GPR137C functional roles in prostate cancer, the top 100 expression-related genes in TCGA-PRAD and GTEx-prostate tissues were identified. Then, we found that the Pearson’s correlation coefficient (PCC) between GPR137C and genes in both PRAD and prostate was approximately 0.5 (**Figure 4A**). However, the top 100 expression-related genes for GPR137C in PRAD and prostate had no identical name (**Figure 4A**). Furthermore, the GPR137C high expression group had more TP53, OTOF, FAM214A, ROBO2, SACS, CSMD3, and KMT2C mutations when compared with the GPR137C low expression group in PRAD (**Figure 4B**). These GPR137C related genes in PRAD were utilized to perform enriched functional analysis. Then, we found that GPR137C has main functions on cell cycle processes, energy metabolism, and location in lysosome membrane (**Figure 4C**). Meanwhile, GPR137C was probably related to prostate cancer progress by some famous pathways, i.e., p53, FoxO, and TGF-beta signaling pathways (**Figure 4D**).

GPR137C was associated with pathways for tumor genomic heterogeneity and cell stem, such as p53 and TGF-beta signaling pathways. Then, the relationship between the expression of GPR137C and PRAD genomic heterogeneity was explored based on four items, i.e., neoantigen (NEO), loss of heterozygosity (LOH), homologous recombination deficiency (HRD), and ploidy. Meanwhile, the relationship between the expression of GPR137C and PRAD tumor cell stem was explored based on three items, i.e., differentially methylated probes-based (DMPss), DNA methylation-based (DNAss), and enhancer elements/DNA methylation-based (ENHss). Then, we found that the GPR137C expression level was positively linked to NEO, LOH, HRD, ploidy, DMPss, DNAss, and ENHss in PRAD (**Figures 4E, F**; **Supplementary Figure S5**). These results suggested that the expression of GPR137C was positively correlated with tumor genomic heterogeneity and stemness of PRAD.

Genomic heterogeneity provides the foundation for cancer stem cells (CSCs) and adaptive subclones. CSCs gain survival advantages by remodeling the microenvironment, while selective pressures from the microenvironment, in turn, drive the evolution of heterogeneity and stemness (42). This complex network not only poses a major challenge in cancer therapy but also offers direction for developing multi-target combination therapies. Additionally, neoantigen load was also associated with higher content of CD8 T cells, macrophages, and CD4 memory T cells in multiple tumor types. Therefore, combined immunotherapy and targeting GPR137C in the treatment of PRAD may be more helpful for patients.

### GPR137C functional roles in prostate cancer tumor microenvironment

**Figure 5:**
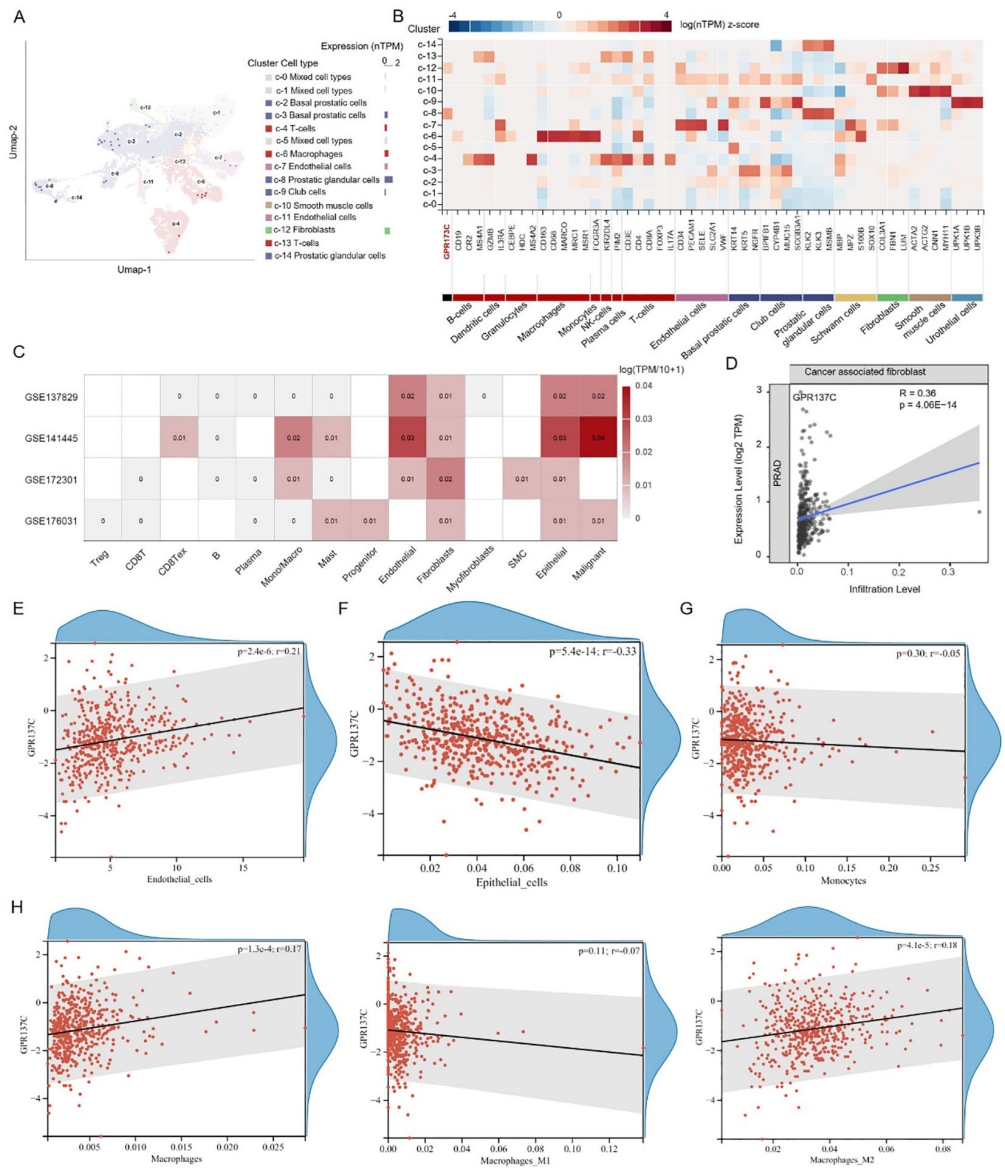
GPR137C affects tumor microenvironment of prostate cancer. **(A)** In prostate tissues, GPR137C expression level in different cell types. **(B)** The GPR137C and marker genes of individual cell-type expression in prostate. **(C)** In prostate cancer tissues, GPR137C expression level in different cell types. The correlation coefficient between GPR137C expression level and infiltration level of cancer-associated fibroblasts **(D)** /endothelial cells **(E)** /epithelial cells **(F)** /monocytes **(G)** /macrophage **(H)**. Macrophages are divided into three parts, i.e., macrophage, M1 macrophage, and M2 macrophage, to calculate correlation coefficients.

GPR137C was found to be highly expressed in prostatic glandular cells and fibroblasts in prostate normal tissues based on data in Human Protein Atlas (HPA) database (**Figures 5A, B**). Then, according to the Gene Expression Omnibus (GEO) database, four prostate cancer signal cell sequencing datasets were downloaded, i.e., GSE137829, GSE141445, GSE172301, and GSE176031. In PRAD tissues, GPR137C was found to be not only highly expressed in malignant cells and fibroblasts but also highly expressed in macrophage, monocytes, epithelial cells, and endothelial cells (**Figure 5C**). These results suggested that GPR137C was highly expressed in various cell types in cancer tissues when it was compared with normal tissues.

According to TCGA-PRAD data, it was found that GPR137C promotes infiltration levels of cancer-associated fibroblast (R = 0.36, p = 4.06E−14) and endothelial cells (R = 0.21, p = 2.40E−06) (**Figures 5D, E**). For epithelial cells, although GPR137C was highly expressed, it did not enhance their infiltration level (**Figure 5F**). However, GPR137C expression level was positively linked to the expression for marker genes of epithelial mesenchymal transformation (EMT) (CDH1, CDH12, VIM, SNAI1, ZEB1, and ZEB2) in PRAD (R = 0.17, p = 4.80E−04) (**Supplementary Figure S6**). This result suggested that the high expression of GPR137C in the epithelial cell probably activated EMT, promoting cancer development. For monocytes and macrophage cells, the GPR137C expression was only positively relative to infiltration levels of macrophages (**Figures 5G, H**). Furthermore, GPR137C only facilitated the M2 macrophage infiltration level and not M1 macrophage in PRAD tissues (**Figure 5H**). These results suggested that high GPR137C expression could affect tumor microenvironment, influencing the progress of prostate cancer.

### GPR137C relation drugs prepared for PRAD patients

**Figure 6:**
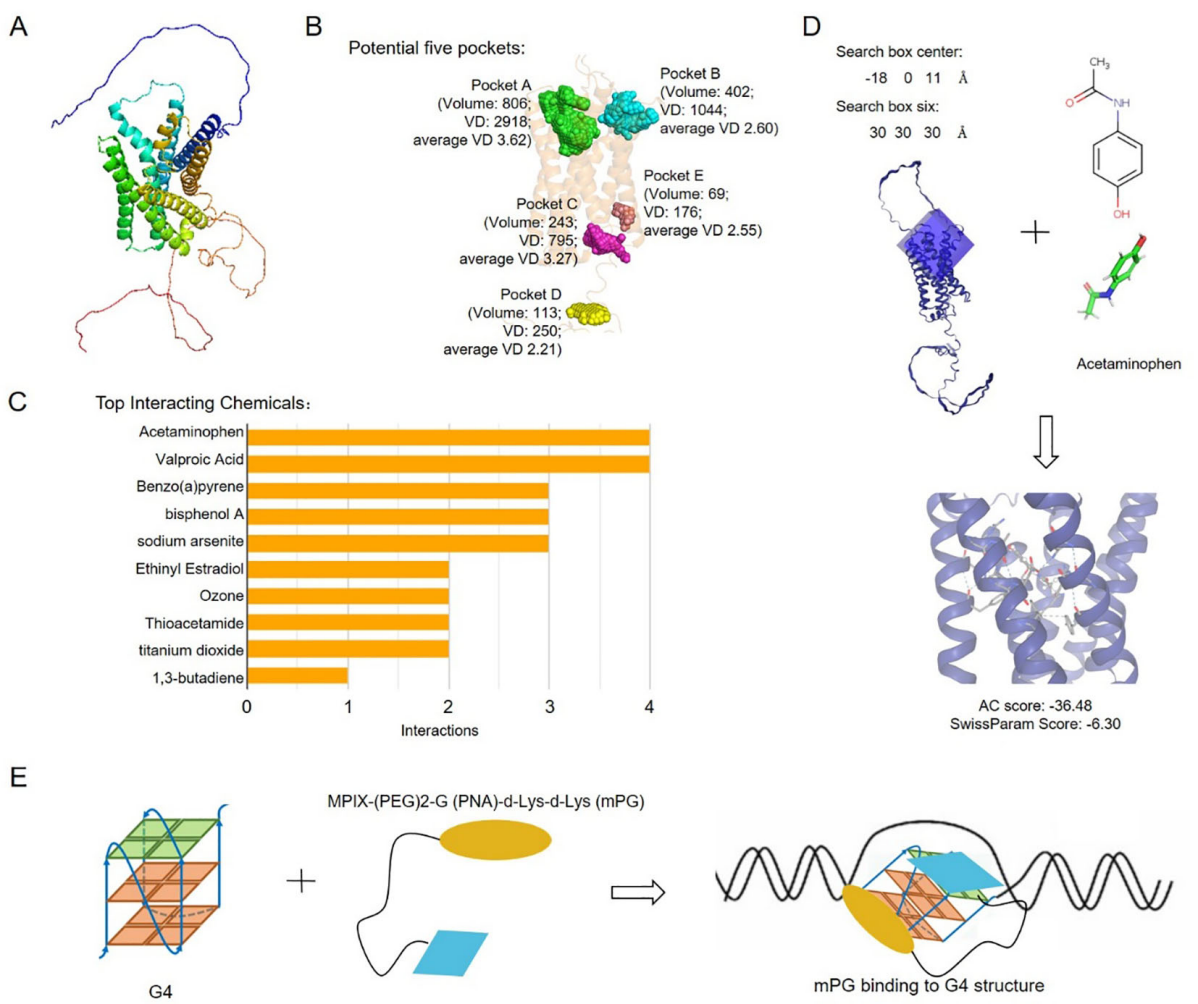
Potential anti-tumor drugs based on GPR137C structure. **(A)** GPR137C structure predicted by AlphaFold3. **(B)** Potential binding pockets of ligands for GPR137C. **(C)** Top 10 interacting chemicals for GPR137C. **(D)** The complex structures for GPR137C and potential drug acetaminophen. **(E)** The schematic drawing complex of MPIX-(PEG) 2-G (PNA)-d-Lys-d-Lys (mPG) and G4 in GPR137C promoter.

To explore potential drugs for PRAD, three-dimensional structure and potential pockets of GPR137C were predicted by Alpha Fold and POCASA software (37, 38). The five pockets of GPR137C were identified based on three-dimensional structure (**Figures 6A, B**). The best one (pocket A) in five pockets was distributed in the N-terminal of GPR137C based on volume depth (VD) values (**Figure 6B**). Meanwhile, potential chemicals for GPR137C were explored by comparative toxicogenomics database. The top 10 interacting chemicals are shown in **Figure 6C**. The top 2 interacting chemical for GPR137C were acetaminophen and valproic acid (**Figure 6C**).

The molecule acetaminophen was then docked with GPR137C by SwissDock software. The complex structure for acetaminophen and GPR137C with a low attracting cavities (AC) score is shown in **Figure 6D**. There were many interactions between acetaminophen and GPR137C. These results indicated that acetaminophen probably acts as a ligand for GPR137C. Furthermore, MPIX-(PEG) 2-G (PNA)-d-Lys-d-Lys (mPG) was a known G4 inhibitor (21). The mPG could be utilized to inhibit G4 formation in the promoter of GPR137C, which induced inhibition GPR137C expression in PRAD tissues (**Figure 6E**). These results suggested that acetaminophen and mPG could be act as potential drugs for PRAD patients.

## Discussion

Since there is a rapid increase in prevalence and mortality for PRAD, it remains one of the leading cancers in the world and an important global healthcare problem for humans (6). PRAD terminal patients are largely threatened with unfavorable clinical prognosis owing to metastasis, drug resistance, and side effects (5). Meanwhile, oGPCRs were known to act as potential biomarkers or pharmacological targets for many cancers (16, 43). In this study, an orphan GPCR, GPR137C, was found closely affiliated to PRAD occurrence, development, and prognosis. Furthermore, G4 plays an important role in gene regulation and is associated with cancer progression (44). The presence of G4 structure is tightly associated with CGI hypomethylation in the human genome, and G4 structure probably sequesters the DNMT3A-CD resulting in hypomethylation and activation of oncogene transcription (41). Then, we identified G4 structures in the promoter of GPR137C, and it has a negative relationship with methylation level in the promoter for GPR137C. Therefore, the G4 structure probably prevents DNMT3A-CD proper binding to DNA, resulting in hypomethylation, which leads to transcriptional activation of GPR137C. In this study, GPR137C could be activated in the PRAD tumors by G4 formation in the promoter, which probably is a novel de-orphanization/activation method.

GPR137C, a lysosome-localized G-protein-coupled receptor-like protein with unknown function, may regulate MTORC1 complex translocation to lysosomes (45). Here, we found that a higher expression of GPR137C (cancer-progress related isoform: ENST00000321662.10) in tumors means a poorer prognosis of prostate cancer. In the enriched analysis of GPR137C-related genes in PRAD, its functions were found to be probably related to cell cycle and energy metabolism. Meanwhile, its expression was positively correlated with tumor heterogeneity and stemness in PRAD tissues. It promotes prostate cancer progress probably by cancer-related pathways, i.e., p53, FoxO, and TGF-beta signaling pathways. Furthermore, GPR137C was found to promote infiltration levels of cancer-associated fibroblast, endothelial cells, and M2 macrophages. For epithelial cells, it probably activated epithelial mesenchymal transformation in PRAD tissues. These results suggested that GPR137C could affect tumor microenvironment and influencing the progress of prostate cancer by p53 and TGF-beta-related pathways. Hence, GPR137C could act as a potential biomarker for PRAD patients.

By computer virtual filtering based on three-dimensional structure, acetaminophen, valproic acid, and MPIX-(PEG) 2-G (PNA)-d-Lys-d-Lys (mPG) probably could be utilized to inhibit the development of PRAD patients. Previous studies had been indicated that acetaminophen use has been associated with blunted vaccine immune responses (46). It could act as a potential suppressor of antitumor immunity (46). These results were consistent with our findings of GPR137C. Acetaminophen acts on antitumor immunity probably based on its target GPR137C functions. Integrated analysis of tumor genomic heterogeneity, stemness, and the immune microenvironment revealed that combined targeted therapy against GPR137C and immunotherapy may offer greater clinical benefits for PRAD patients.

In summary, our findings suggest that GPR137C plays a critical role in immune infiltration and may serve as a potential diagnostic and prognostic molecular marker for PRAD. Additionally, GPR137C likely regulates several key pathways involved in energy metabolism, cell cycle, tumor heterogeneity, and stemness in PRAD. Moreover, G4 in promoter probably is a novel de-orphanization/activation method for oGPCRs. Furthermore, acetaminophen, valproic acid, and mPG probably could be utilized to inhibit GPR137C. However, the regulatory mechanisms of GPR137C in the gastric cancer tumor immune microenvironment and the targeting specificity and clinical efficacy of the potential drugs require further investigation and discovery through our future research efforts. Therefore, we hope that our results will provide novel insights into the activation of oGPCRs and the development of immunotherapeutic drugs, aid clinicians in selecting appropriate treatments for PRAD patients, and contribute to the identification of biomarkers that can predict the survival of PRAD patients more accurately.

## Conclusion

In this study, we investigated the relationship between GPR137C expression and G4 structure, immune infiltration, tumor genetic heterogeneity, tumor stemness, protein interactions, and genomic alterations and analyzed GPR137C DNA methylation and small molecule drugs in PRAD. Our findings suggest that GPR137C is a promising independent prognostic factor and is closely associated with immune infiltration level and cancer progression in PRAD. G4 in the promoter could act as one of de-orphanization/activation methods for oGPCRs. This study comprehensively reveals the role of GPR137C in PRAD and its potential as a promising novel marker.

## Data Availability

The original contributions presented in the study are included in the article/supplementary material. Further inquiries can be directed to the corresponding authors.
