# Supplementary material for: Investigation of GPR137C as a promising novel marker for the progression of prostate cancer through G4 screen and bioinformatics analyses
Source: Front Immunol. 2025 May 30;16:1576835. doi: 10.3389/fimmu.2025.1576835 (PMC12162501; doi:10.3389/fimmu.2025.1576835)
Supplement: Supplementary file 1 [file DataSheet1.docx]

# Investigation of GPR137C as a promising novel marker for the progression of prostate cancer through G4 screen and bioinformatics analyses

Yue Hou^1#^, Haowen Lu^2#^, Saisai Chen^2^, Likai Mao^2^, Xuan Huang^3*^, Feng Xu^4*^, Chuanjun Shu^5#*^

^1^Military Medical Innovation Center, Fourth Military Medical University, Xi’an 710032, China.

^2^Department of Urology, Affiliated Zhongda Hospital of Southeast University, Nanjing, Jiangsu 210009, China.

^3^Reproductive Medical Center, Jinling Hospital Affiliated to Medical School of Nanjing University, Nanjing, Jiangsu 210002, China.

^4^Department of Urology, Jinhu County People's Hospital, Huai an, Jiangsu 210002, China.

^5^Department of Bioinformatics, School of Biomedical Engineering and Informatics, Nanjing Medical University, Nanjing 211166, China.

#These authors contribute equally to this study.

*Corresponding author: Email: Chuanjun Shu: [chuanjunshu@njmu.edu.cn](mailto:chuanjunshu@njmu.edu.cn)；Feng Xu: jhxfzj@126.com; Xuan Huang: huangxuan1670@163.com.


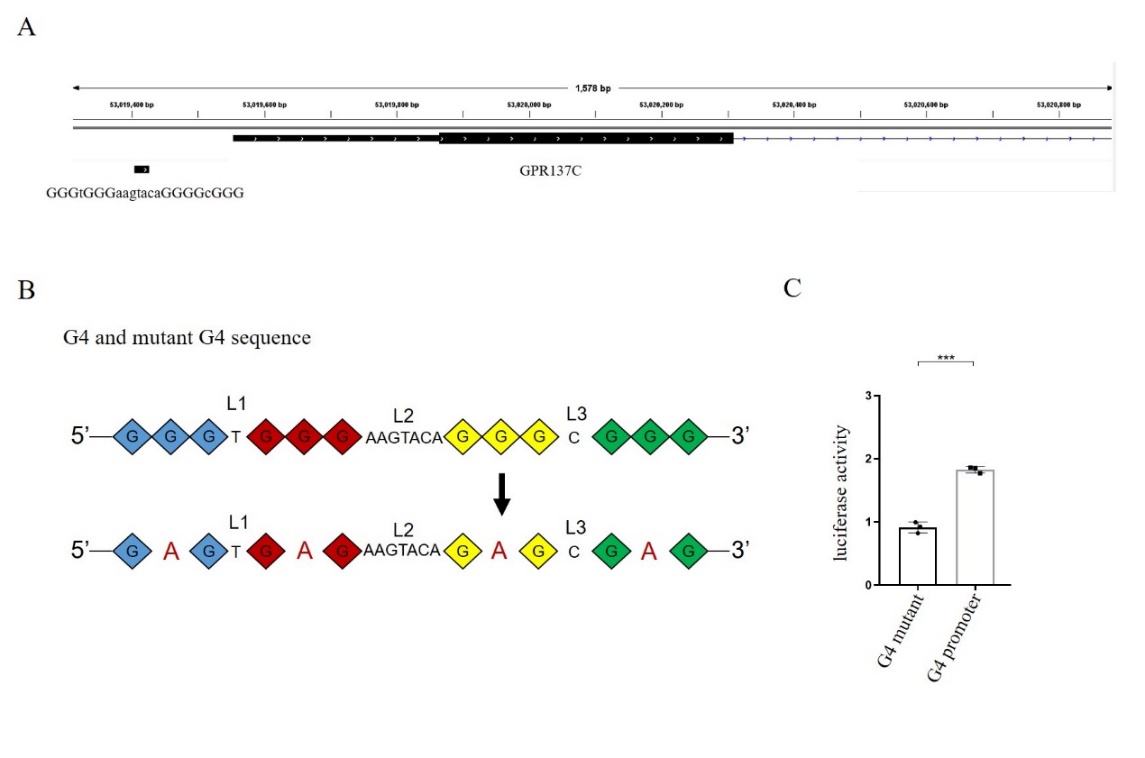


**Figure S1. G4 enhances expression level of GPR137C.** (A) G4 sequences in promoter of GPR137C. (B) G4 and mutant G4 sequence. (C) Luciferase activity for G4 and G4 mutant.


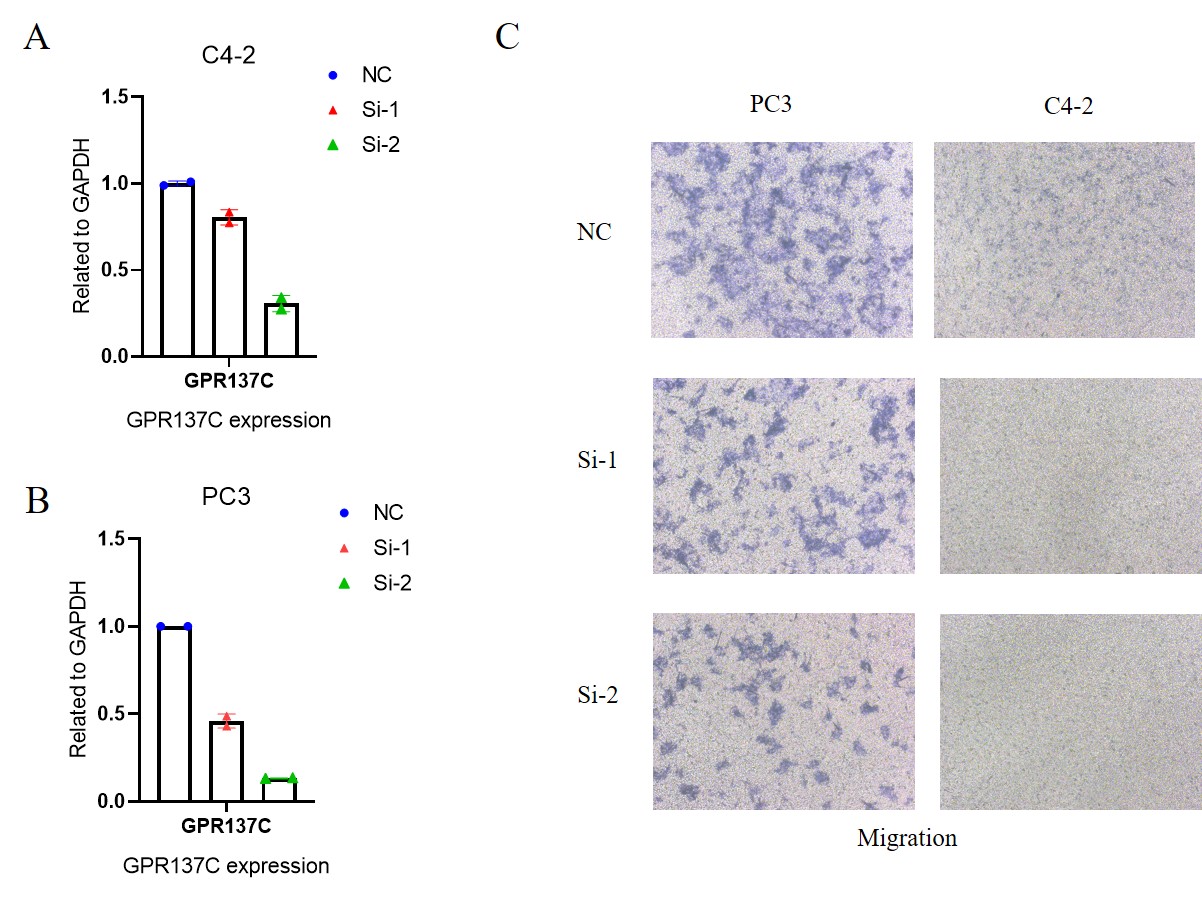


**Figure S2. GPR137C promotes migration of prostate cancer cells.** The small interfering RNA influence for GPR137C expression in C4-2 (A) and PC3 (B). (C) Transwell analysis results for si-GPR137C and NC (normal control).

A


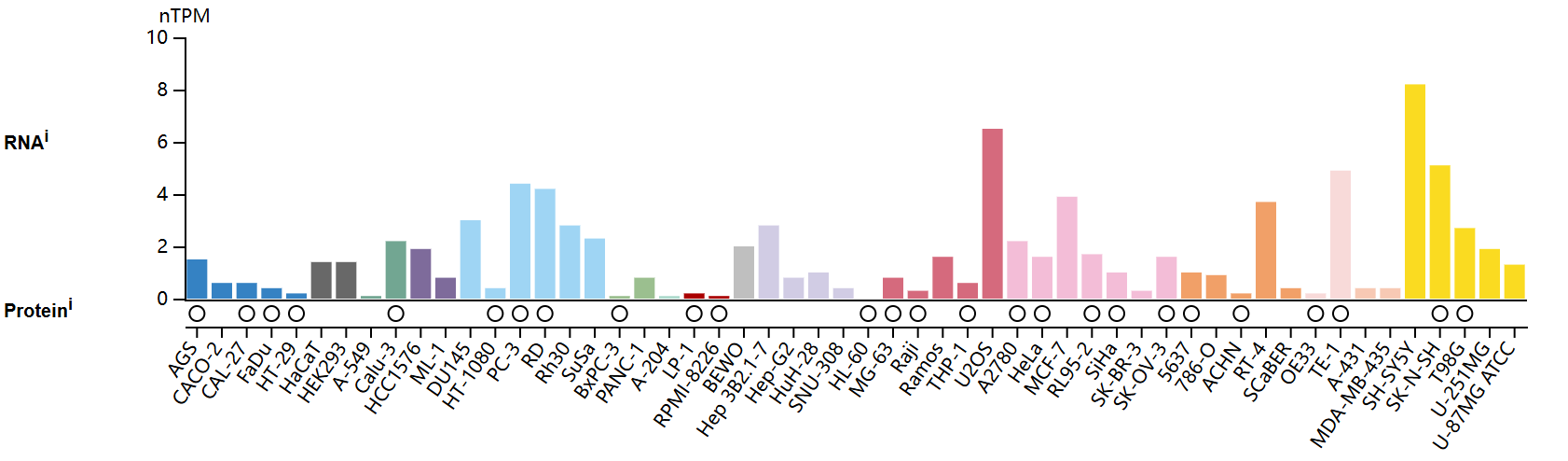


B


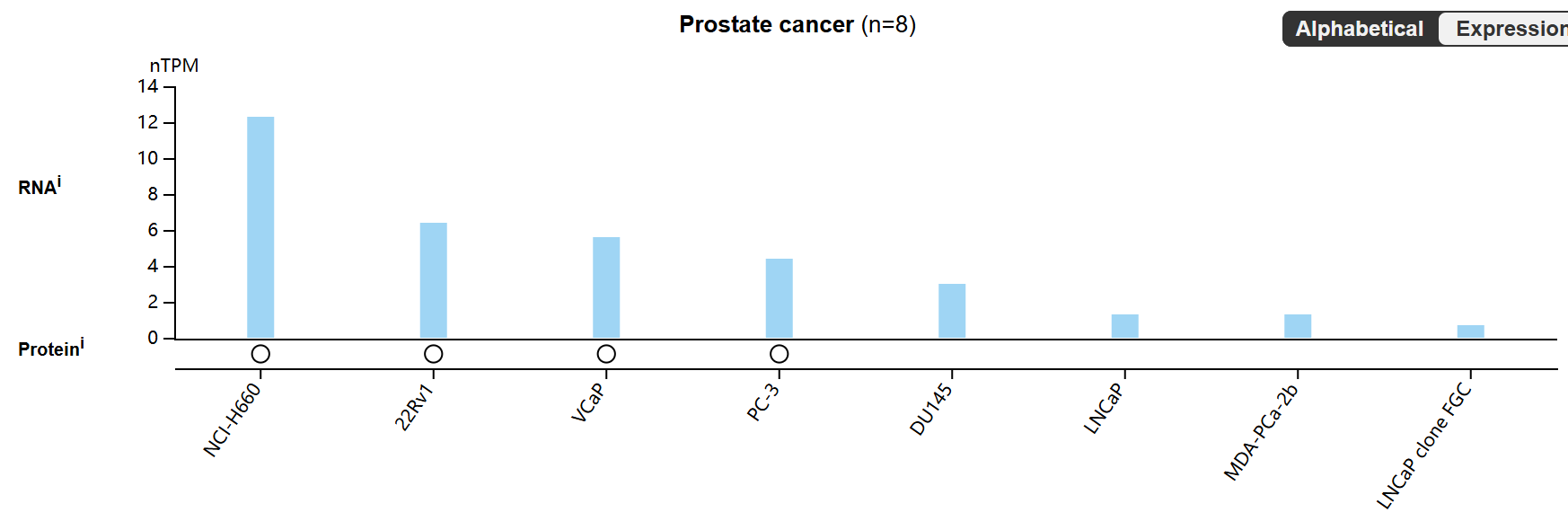


**Figure S3. GPR137C expression in prostate cancer cells based on HPA database.** GPR137C expression in common cell lines (A) and eight types of prostate cancer cells (B).


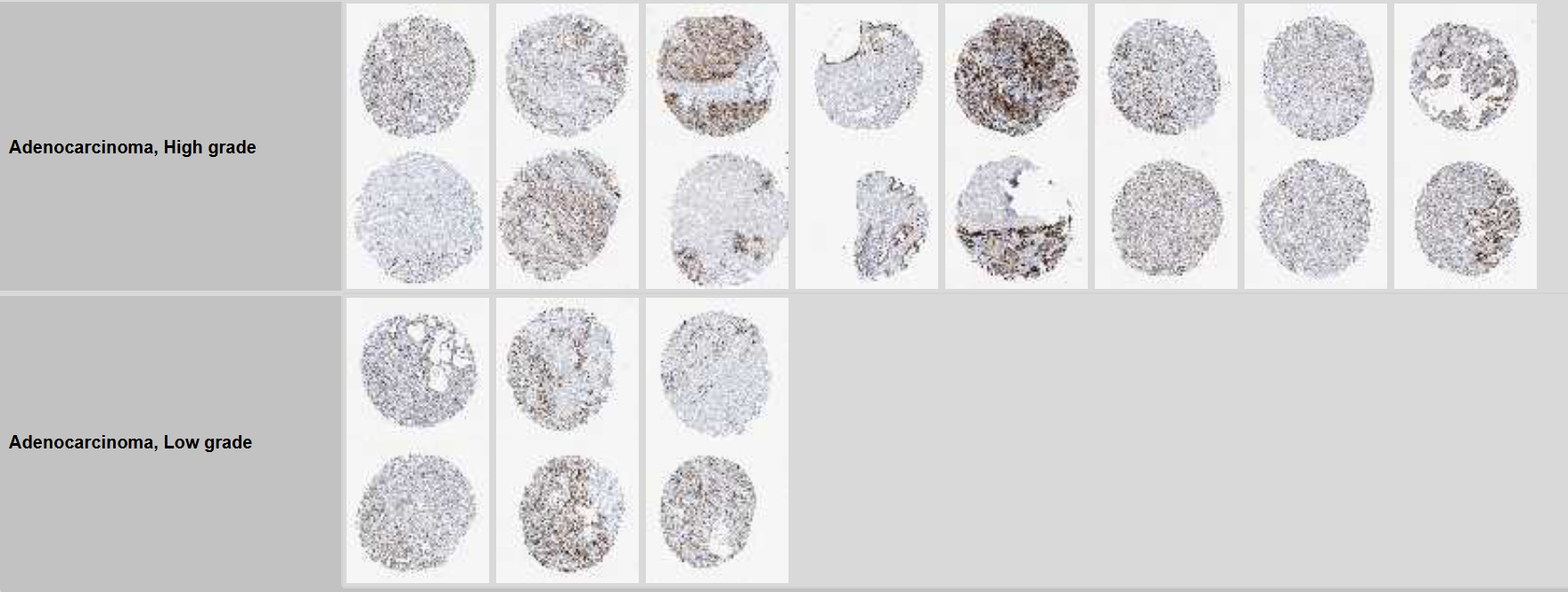


**Figure S4. GPR137C antibody staining in PRAD patients.**


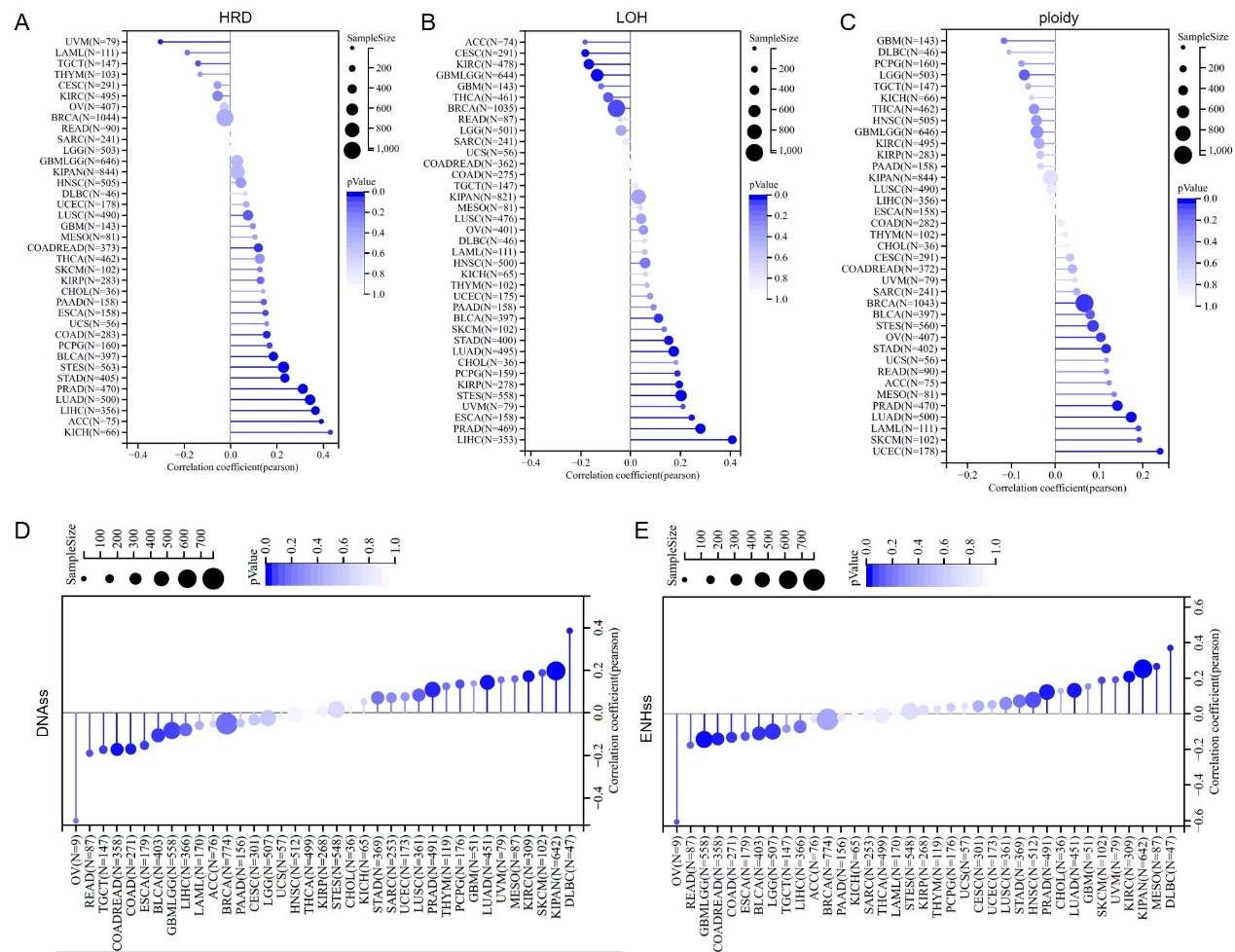


**Figure S5. The correlation coefficient between GPR137C and HRD / LOH / ploidy / DNAss / ENHss.** GPR137C expression level was positively linked to HRD (A), LOH (B), ploidy (C), DNAss (D), and ENHss (E) in PRAD.


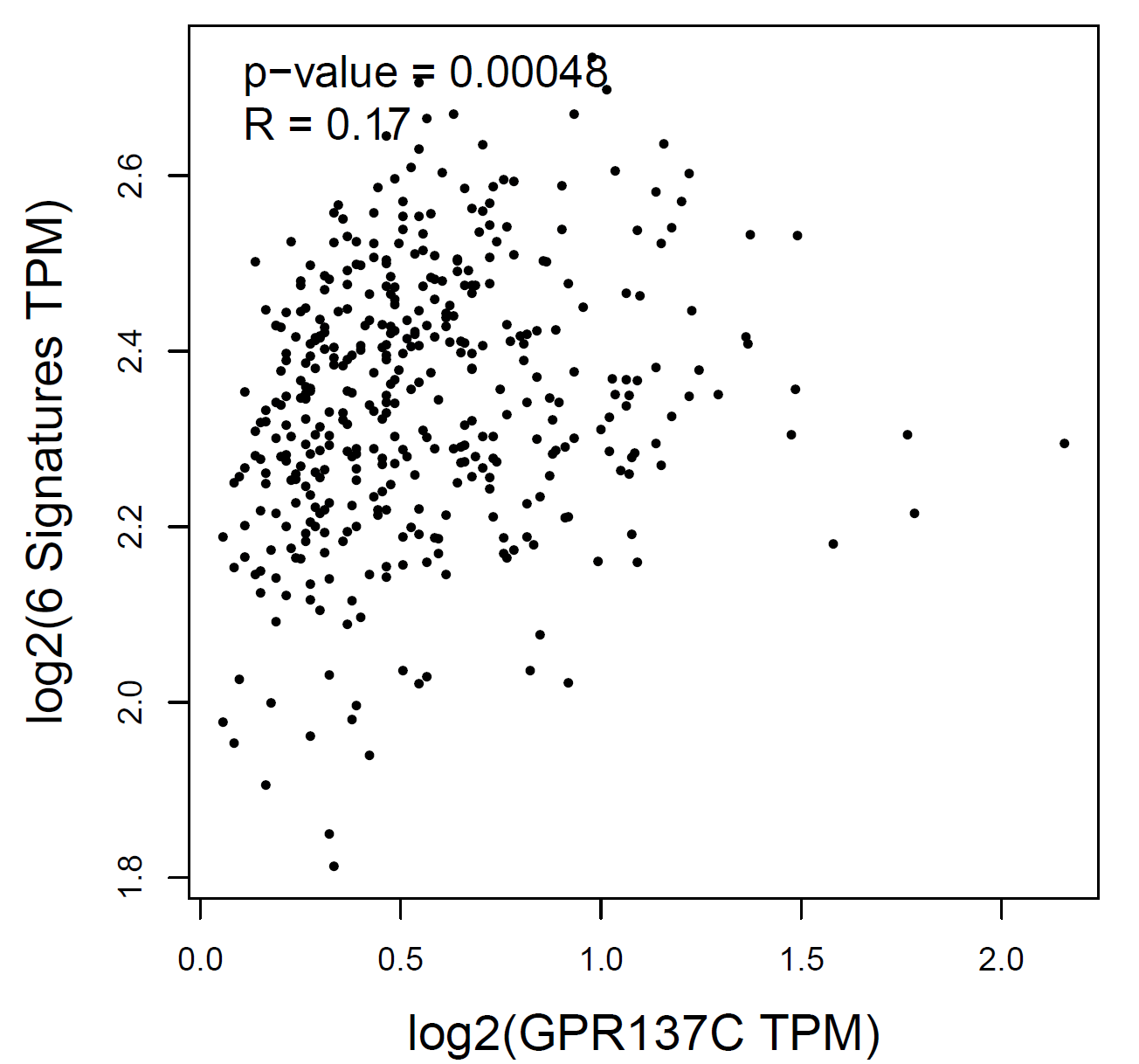


**Figure S6. The correlation coefficient between GPR137C and 6 signature genes of EMT.**
